# An *Arabidopsis thaliana* copper-sensitive mutant suggests a role of phytosulfokine in ethylene production

**DOI:** 10.1093/jxb/erv105

**Published:** 2015-04-23

**Authors:** Tao Wu, Takehiro Kamiya, Hiroko Yumoto, Naoyuki Sotta, Yamaguchi Katsushi, Shuji Shigenobu, Yoshikatsu Matsubayashi, Toru Fujiwara

**Affiliations:** ^1^Department of Applied Biological Chemistry, Graduate School of Agricultural and Life Sciences, The University of Tokyo, Bunkyo-ku, Tokyo 113-8657, Japan; ^2^Key Laboratory of Biology and Genetic Improvement of Horticultural Crops (Northeast Region, Ministry of Agriculture), Horticultural College, Northeast Agricultural University, 59 Mucai Street, Harbin 150030, China; ^3^National Agriculture and Food Research Organization, Institute of Floricultural Science, 3-1-1 Kannondai, Tsukuba, Ibaraki 305-8666, Japan; ^4^National Institute for Basic Biology, Okazaki 444-8585, Japan; ^5^Division of Biological Science, Graduate School of Science, Nagoya Universisy, Chikusa-ku, Nagoya 464-8602, Japan

**Keywords:** Cu, deficiency, ethylene, PSK, root elongation, TPST.

## Abstract

To increase our understanding of the adaptation for copper (Cu) deficiency, *Arabidopsis* mutants with apparent alterations under Cu deficiency were identified. In this report, a novel mutant, *tpst-2*, was found to be more sensitive than wild-type (Col-0) plants to Cu deficiency during root elongation. The positional cloning of *tpst-2* revealed that this gene encodes a tyrosylprotein sulfotransferase (TPST). Moreover, the ethylene production of *tpst-2* mutant was higher than that of Col-0 under Cu deficiency, and adding the ethylene response inhibitor AgNO_3_ partially rescued defects in root elongation. Interestingly, peptide hormone phytosulfokine (PSK) treatment also repressed the ethylene production of *tpst-2* mutant plants. Our results revealed that TPST suppressed ethylene production through the action of PSK.

## Highlight

A new role of phytosulfokine in the regulation of ethylene production was identified.

## Introduction

Cell-to-cell communication plays an important role in the response to developmental and environmental cues in multicellular organisms. In plants, phytohormones (such as auxin, cytokinins, and ethylene) play critical roles in long-distance cell-to-cell interactions. In addition, secreted peptides [e.g., phytosulfokine (PSK), plant peptide containing sulfated tyrosine 1 (PSY1), and root growth factor] have recently emerged as a novel type of plant hormone (Matsubayashi and Sakagami, 1996; Matsubayashi, 2011, 2014; Yamada and Sawa, 2013). These peptides function as intercellular signalling molecules that coordinate and specify cellular functions in plants. Some of the secreted peptide hormones undergo complex posttranslational modifications mediated by specific enzymes that recognize particular sequences of multiple target peptides (Matsuzaki *et al.*, 2010). Protein tyrosine sulfation is a form of posttranslational modification and is mediated by tyrosylprotein sulfotransferases (TPSTs). The transfer of sulfate from 3′-phosphoadenosine 5′-phosphosulfate to the hydroxyl group of peptidyl tyrosine residues is catalysed by TPSTs to form a tyrosine O4-sulfate ester (Bettelheim, 1954; Huttner, 1982). In animals, TPSTs are located in the trans-Golgi network and distributed in various tissues and cells. Several of these enzymes play important roles in inflammation, haemostasis, and immunity (Moore, 2003). However, plants have evolved structurally different TPSTs from their animal counterparts, which are critical for the biological activities of the peptide hormones PSK and PSY1 in plants (Matsubayashi, 2011). A recombinant version of an *Arabidopsis thaliana TPST*, At1g08030, expressed in yeast catalysed tyrosine sulfation of both PSK and PSY1 precursor polypeptides *in vitro* (Komori *et al.*, 2009). *At*TPST, a type I transmembrane protein localized in *cis*-Golgi, is expressed throughout the plant body, with the highest expression observed in the root apical meristem (Komori *et al.*, 2009). Moreover, TPST is associated with auxin and brassinosteroid, which exhibit diverse roles for sulfated peptides in plant growth and development (Zhou *et al.*, 2010; Hartmann *et al.*, 2013). Although the role of the tyrosine sulfation motif in peptides remains unclear, characterizing peptide signalling is important for future plant research.

Copper (Cu) is an essential micronutrient for the growth and development of plants. Many metabolic processes, such as photosynthesis, mitochondrial electron transport, respiration, reactive oxygen metabolism, cell wall remodelling, and ethylene perception, also require Cu as an essential cofactor (Pilon *et al.*, 2006; Burkhead *et al.*, 2009). The Cu contents of plants vary among species, and environmental Cu conditions affect these Cu contents (Cohu and Pilon, 2007). The Cu content in plants typically ranges from 2 µg g^−1^ to 50 µg g^−1^ dry weight (DW) (ppm), and a Cu content of 6 µg g^−1^ is considered adequate for the normal growth and development of plants (Epstein and Bloom, 2005). However, when Cu content is lower than 5 µg g^−1^ DW or higher than 20 µg g^−1^ DW, symptoms of deficiency or toxicity appear (Marschner, 1995; Burkhead *et al.*, 2009). Cu deficiency (-Cu) varies by agricultural soil types or crops; organic soil, including peat soils, and mineral soils with high levels of organic matter (6–10%) are most likely to be deficient in available Cu. -Cu develops on organic soils mainly because of Cu binding to organic matter (Marschner, 1995; Shorrocks and Alloway, 1988). Symptoms of -Cu in plants include reduced growth rates, leaf bleaching, pollen infertility, and a reduction in seed set and yield (Marschner, 1995).

In response to -Cu, plants control Cu homeostasis by regulating Cu uptake and allocation (Puig and Thiele, 2002; De Freitas *et al.*, 2003). One of the mechanisms activated by *Arabidopsis* plants in response to -Cu is elevating Cu uptake. In *Arabidopsis*, the expression of some Cu transporter genes is upregulated in response to -Cu (Wintz *et al.*, 2003; Mukherjee *et al.*, 2006). The genes for Cu transporters zinc-regulated transporter iron-regulated transporter protein 2 (*ZIP2*) and *ZIP4* are upregulated under -Cu (Wintz *et al.*, 2003). Ferric reductase oxidase 3 (*FRO3*) expression is also induced in roots and shoots by Cu limitation (Mukherjee *et al.*, 2006). In addition, under Cu-deficient conditions, copper chaperone (*CCH*) may be involved in mobilizing limited Cu among tissues during senescence (Mira *et al.*, 2002). The production of *CCH* is also upregulated under -Cu. Another mechanism of plant response to -Cu is modifying Cu allocation for improved Cu economy, which has been well characterized in *Chlamydomonas reinhardtii* (Merchant *et al.*, 2006). However, *A. thaliana* plants have a Cu allocation pattern different from that of *C. reinhardtii*. In *A. thaliana*, the expression patterns of copper/zinc superoxide dismutase1 (*CSD1*) and *CSD2*, as well as copper chaperone for SOD (*CCS*), show similar responses to the availability of Cu; i.e., these genes are downregulated under -Cu (Wintz *et al.*, 2003; Abdel-Ghany *et al.*, 2005). Given that *CSD1* and *CSD2* are downregulated under -Cu, ferric superoxide dismutase 1 (*FSD1*) becomes active, and the function of Cu/ZnSOD is replaced by FeSOD. Cu is then stored for the most essential functions under -Cu conditions (Abdel-Ghany *et al.*, 2005; Cohu and Pilon, 2007). Identifying Cu-microRNAs whose expression is upregulated by low Cu increased our understanding of the mechanism of Cu homeostasis. *miR398* regulates the mRNA stability of major Cu-containing proteins, such as CSD1, CSD2, and COX5b-1, under Cu-limited conditions. *miR398* can directly regulate *CSD* mRNA degradation under -Cu (Yamasaki *et al.*, 2007). In addition to *miR398*, three microRNAs, namely, *miR397*, *miR408*, and *miR857*, target genes that encode Cu-containing proteins (Abdel-Ghany and Pilon, 2008). -Cu activates *miR398b* and *miR398c* transcriptions via these GTAC motifs in *A. thaliana*, and the GTAC motif is sufficient for the response to -Cu (Yamasaki *et al.*, 2009). The sequence analysis revealed that *A. thaliana* transcription factor squamosa promoter binding protein-like 7 (*SPL7*) is a candidate homologue of *C. reinhardtii* CRR1 protein, which is involved in Cu homeostasis in *Chlamydomonas* (Cardon *et al.*, 1999; Kropat *et al.*, 2005). Further analysis indicated that *SPL7* is a conserved central regulator of Cu-deficiency response in plants. *SPL7* can bind directly to GATC motifs in *miR398* promoter (Yamasaki *et al.*, 2009). In addition, some genes that encode Cu transporters (*COPT1*, *COPT2*, *ZIP2*, and *YSL2*), Cu chaperones (*CCH* and *CCS*), *FRO3*, *FRO4*, *FRO5*, and *FSD1* are also misregulated in *spl7* plants under -Cu (Yamasaki *et al.*, 2009; Bernal *et al.*, 2012). Moreover, many new genes in response to -Cu are regulated by *SPL7* (Yamasaki *et al.*, 2009). Despite the importance of preventing -Cu in plants, our understanding of the mechanisms controlling Cu homeostasis and trafficking inside the cell remains limited.

An attempt is made here to identify mutants with apparent alterations under -Cu to gain further insight into the molecular requirements of Cu homeostasis. In this paper, a novel mutant, *tpst-2*, is described as sensitive to -Cu in root length. This *tpst-2* mutant has a considerably shorter root phenotype under -Cu conditions, whereas a high concentration of Cu (50 μM Cu) can partially recover its root elongation. Cu content of *tpst-2* was lower than that of wild type Col-0. Positional cloning of *tpst-2* revealed that this gene encodes a tyrosylprotein sulfotransferase (TPST). Moreover, *tpst-2* was sensitive to ethylene, and adding AgNO_3_ to *tpst-2* plants could partially rescue the root elongation of the mutant under -Cu. The ethylene production of *tpst-2* mutant was higher than that of Col-0 under -Cu. Moreover, peptide hormone PSK treatment also repressed the ethylene production of *tpst-2* mutant plants. The results obtained in this study reveal that TPST suppresses ethylene production through PSK action.

## Materials and methods

### Plant materials and growth conditions

Ethyl-methanesulfate (EMS)-mutagenized Columbia (Col-0 *gl1-1*) seeds were purchased from Lehle Seeds (Cat. No. M2E-01A-07; Roud Rock TX, USA). The seeds of the homozygous lines of T-DNA insertion SALK_009847 (*tpst-1*) and *35S:AtTPST*/*tpst-1* were kindly provided by Prof. Matsubayashi (Komori *et al.*, 2009). F_1_ seeds used in the allelism test were obtained from a cross between *tpst-1* and *33–4*. *35S:AtTPST/33–4* seeds were obtained from F_3_ of a cross between *35S:AtTPST*/*tpst-1* and *33–4*. The point mutations in *33–4* lines carrying the *35S:AtTPST* gene were detected using CAPS primers with the *Dde*I restriction site (Supplementary Table S1). The seeds were surface-sterilized and sowed on an MGRL growth medium (Fujiwara *et al.*, 1992) solidified with 1.5% gellan gum (Wako Pure Chemical Industries, Ltd., Osaka, Japan) and supplemented with 1% sucrose. After the seeds were incubated for 2 d at 4°C, the plates were placed vertically and the plants were grown at 22°C under a 16h light/8h dark photoperiod. The plants were supplied with high Cu nutrient (50 μM Cu) containing 1.512mM NaH_2_PO_4_·2H_2_O, 0.257mM Na_2_HPO_4_·12H_2_O, 1.5mM MgSO_4_·7H_2_O, 4mM Ca(NO_3_)_2_·4H_2_O, 6mM KNO_3_, 8.6 μM C_10_H_12_FeN_2_NaO_8_·3H_2_O, 10.3 μM MnSO_4_, 50 μM CuSO_4_·5H_2_O, 30 μM H_3_BO_3_, 24nM Na_6_Mo_7_O_24_·4H_2_O, and 130nM CoCl_2_·6H_2_O (Fujiwara *et al.*, 1992). The plants were also grown under a -Cu condition, in which no Cu was supplied in the medium.

### Mutant selection and genetic mapping

EMS-mutagenized M_2_ seeds [50 seeds/plate × 20 plates per batch; a total of 26 batches (ca. 26,000 seeds)] was screened and grown on -Cu (MGRL without CuSO_4_) medium. The plants with short roots were transferred to MGRL with 50 µM CuSO_4_ (50 Cu), and the position of the root tips was marked. After 5 d, the plants with root elongations recovered by 50 Cu were transferred to the -Cu medium. After 5 d, the plants with terminated or slowed root elongations were selected. M_3_ seeds were obtained and then sown in -Cu and 50 Cu media; the corresponding phenotype was confirmed. After the second screening was performed, the *33–4* mutant was obtained.

In mapping, the *33–4* mutant was crossed with Ler, and F_2_ seeds were obtained. Genomic DNA was isolated from F_2_ plants exhibiting a mutant phenotype under -Cu condition; the gene was mapped using simple sequence length polymorphism (SSLP) markers (Sakamoto *et al.*, 2011). After rough mapping was completed, the following primer pairs were used in the final step of the mapping: F24B9-F (5′-GGGGTCATTTGTGATCGAAG-3′) and F24B9-R (5′- TAA ATCACGTATGCCGCTCA-3′); F22O13-F (5′-TACGCAATGAG CCCTCAAAT-3′) and F22O13-R (5′- CTTGCATTGGGTTCA TTCCT-3′). Whole-genome SOLiD sequencing was also performed as described by Tabata *et al.* (2013).

### Quantitative reverse transcription PCR analysis

Total RNA was prepared from the plants using an RNeasy plant mini kit (Qiagen, Valencia, CA, USA) and treated with RNase-free DNase (Qiagen). Total RNA was reverse-transcribed to prepare cDNA by using a PrimeScript RT reagent kit, diluted 2-fold, and used for real-time PCR analysis with a Thermal Cycler Dice system (Takara, Ohtsu, Japan) and SYBR Premix Ex *Taq* II (Takara). The sequences of the primers used in this study are provided in Supplementary Table S1. The primers used for elongation factor 1a (*EF1a*, internal standard) were described previously by Takano *et al.* (2006).

### Analysis of Cu concentration

Whole roots and shoots of Col-0 and *tpst-2* plants grown on -Cu or 50 Cu conditions for 10 d were harvested; samples were washed with distilled water thrice, dried at 65°C, and digested with concentrated nitric acid. The Cu concentration was then determined using inductively coupled plasma mass spectrometry (SPQ9700; SII, Chiba, Japan) as described previously (Takano *et al.*, 2001).

### Determination of sensitivity to Cu, AgNO_3_, 1-aminocyclopropane-1-carboxylic acid, and PSK

The seeds were sown on a medium containing various concentrations of Cu and combined Cu and 1 µM AgNO_3_, 1 µM 1-aminocyclopropane-1-carboxylic acid (ACC), or 10nM PSK to determine the sensitivity of *tpst-2* mutant plants to AgNO_3_ and ACC. The root length of 10-day-old plants was determined using ImageJ (http://rsb.info.nih.gov/ij/).

### Root length measurements

Sterilized seeds were incubated at 4°C for 2 d; afterwards, the plates were placed vertically and the plants were grown at 22°C in a 16h light/8h dark photoperiod. Root length was measured using ImageJ. Data are presented as means ± standard deviation. At least 30 seedlings were subjected to statistical analysis in each case. Tukey’s test was conducted for statistical analysis (*P* < 0.05).

### Ethylene measurements

Seedlings (approximately 30 per vial) were grown in 22-mL gas chromatograph vials containing 3mL of MS medium or MGRL medium with various concentrations of Cu. The vials were flushed with hydrocarbon-free air and then capped at indicated times. The amount of accumulated ethylene was measured using a GC-14B gas chromatograph (Shimazu Corporation, Kyoto, Japan) equipped with an alumina column and a flame ionization detector. Ethylene peaks were quantified using Sic 480 II DataStation software (System Instruments Co., Ltd., Tokyo, Japan) and compared with a 10.06 μL/L ethylene standard. Ethylene production was normalized in terms of the number of seedlings in each vial and the time between capping and sampling. The basal levels of ethylene from Col-0, *tpst-1*, and *tpst-2* were determined from three independent experiments of at least three replicates.

## Results

### Isolation and phenotypic characterization of a Cu-deficiency-sensitive mutant, 33–4

**Fig. 1.**
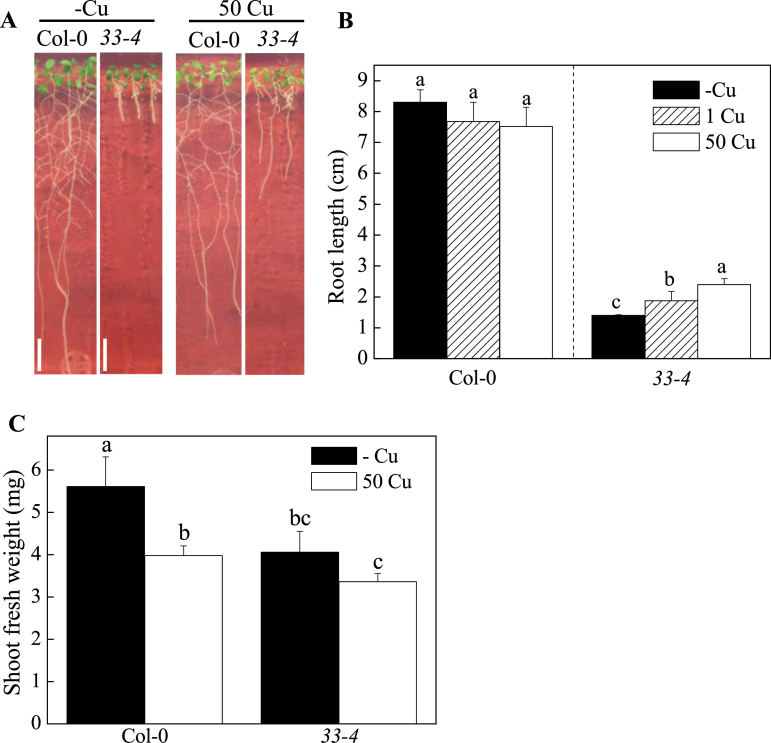
Phenotypes of *Arabidopsis 33–4* mutants in response to Cu conditions. (A) Col-0, *33–4* seedlings grown in -Cu or 50 Cu for 10 d. Bar = 1cm. (B) Root lengths of Col-0 and *33–4* seedlings. The primary root lengths of 10-day-old seedlings grown in -Cu, 1 Cu (normal medium), or 50 Cu are expressed as means ± SE (n = 10). Letters represent significant differences at the 0.05 level based on Tukey’s test. (C) Shoot fresh weight of Col-0 and *33–4* seedlings. The shoot fresh weights of 10-day-old seedlings grown in -Cu or 50 Cu are expressed as means ± SE (n = 10). Letters represent significant differences at the 0.05 level based on Tukey’s test.

In the mutant screening, mutated seeds were repeatedly grown between -Cu medium and 50 Cu medium, to obtain Cu-deficiency-specific sensitive mutant. EMS-mutagenized M_2_ seeds (*n* = 26,000 seeds in total, Col-0 *gl1-1*) were sown onto -Cu medium to identify novel mutants under -Cu condition. Plants with short roots were selected and transferred to 50 Cu medium. The concentration of Cu in the standard medium was 1 µM. After 5 d, the plants with recovered root elongation were transferred to -Cu medium. After another 5 d, the plants with halted or reduced root elongation were selected and M_3_ seeds were harvested from individual plants. M_3_ seeds were sown onto -Cu and 50 Cu media and the lines with differential growth were selected. After this screening was completed, one mutant, namely *33–4*, was obtained (Fig. 1A). The measured root lengths confirmed that root elongation was impeded (Fig. 1B). Under -Cu condition, the root lengths of Col-0 and *33–4* seedlings were 8.28±0.11 and 1.42±0.11cm, respectively. Under 50 Cu condition, the root lengths of Col-0 and *33–4* were 7.0±0.33 and 2.6±0.07cm, respectively. These data revealed that *33–4* was a Cu-deficiency-sensitive mutant. The shoot phenotype of *33–4* was indistinguishable from that observed in Col-0 under -Cu and 50 Cu conditions (Fig. 1A); however, Col-0 plants yielded heavier fresh shoot weights than the *33–4* mutant under 50 Cu and -Cu conditions (Fig. 1C). In addition, Col-0 plants showed heavier fresh shoot weights under -Cu condition than the plants under 50 Cu condition; by contrast, *33–4* plants showed similar shoot fresh weights under both -Cu and 50 Cu conditions (Fig. 1C).

### Identification of genes responsible for the increased low-Cu sensitivity of the *33–4* mutant

The *33–4* mutant in the Col-0 background was crossed with Ler wild-type plants for genetic analysis and mapping. The resultant F_2_ progeny showed a segregation of the short- and long-root phenotypes. Among 134 plants, 25 exhibited mutant short-root phenotype and 109 displayed wild-type long-root phenotype. This result was not significantly different from the segregation ratio of 1:3 (*P* = 0.09, chi-square test), indicating that *33–4* carries a single recessive mutation responsible for the short-root phenotype. In mapping, genomic DNA was isolated from 200 individual F_2_ plants that exhibited the mutant phenotype under -Cu condition; the mutation was then mapped using SSLP markers. The causal gene was roughly mapped to chromosome 1 and then finely mapped between SSLP markers F24B9 (2.42Mb) and F22O13 (2.78Mb; Supplementary Fig. S1A). This region contained 93 putative genes annotated in TAIR10 (Fig. S1A). To identify point mutations, the genomic DNA of the *33–4* mutant was sequenced using the SOLiD sequencer. Two nucleotide substitutions (SNPs) were identified from the sequencing data. One SNP was found in At1g07910, resulting in an amino acid substitution. Another SNP was found in an intron of At1g08030 (Fig. S1A). At1g08030 is annotated as tyrosyl protein sulfotransferase (*TPST*); a G-to-A transition at nucleotide no. 2491575 of the genomic sequence (the consensus splicing site of fourth intron) was found. This mutation likely disrupted splicing. The phenotype of *tpst-1* mutants has been described previously (Komori *et al.*, 2009); the abnormal root elongation phenotype was similar to the *33–4* mutant.

A mutant line (*tpst-1*, SALK_009847) homozygous to a T-DNA insertion in the *AtTPST* gene was examined (Fig. S1B) to determine whether *TPST* is the causal gene of *33–4*. *tpst-1* and F_1_ plants derived from crosses between *33–4* and *tpst-1* showed the same phenotype as *33–4* under the -Cu conditions; similar to the case of *33–4*, root elongation was partially recovered by treatment with 50 Cu (Fig. S1C). These findings suggest that the phenotypes of *33–4* and *tpst-1* are caused by a mutation in the same locus. Furthermore, the introduction of *35S:AtTPST* to the *33–4* mutant restored the wild-type phenotype under both -Cu and 50 Cu growth conditions (Fig. S1C). These results indicate that the causal gene of *33–4* is indeed *TPST*. In this study, *33–4* is referred to as *tpst-2* hereafter.

### A mutation in *TPST* in *tpst-2* disrupts splicing

The point mutation in *tpst-2* is in the splicing consensus sequence in the fourth intron. The mRNA of *TPST* in *tpst-2* was examined to study the effect of the point mutation on its expression. RT-PCR analysis was performed using primers that amplified the PCR products covering the mutation region (Supplementary Table S1). Based on the sequence information of *TPST*, the PCR product using the designed primers should have been 409bp in length. A single band of the expected size was produced by RT-PCR using the wild-type mRNA. The mRNA of *tpst-2* resulted in two PCR products (Supplementary Fig. S2). The PCR products were then sequenced. The size of the band from the wild type was 409bp; by contrast, the bands from the mutant were 517 and 381bp in length. Based on the sequence, the structure of each transcript was determined (Fig. S2B,C,D). The wild-type transcript yielded the expected structure; by contrast, the transcripts from the mutant showed either a deletion or an insertion of extra sequences. These transcripts were unlikely to produce a wild-type protein.

### Mutation of *TPST* specifically affects the Cu response

**Fig. 2.**
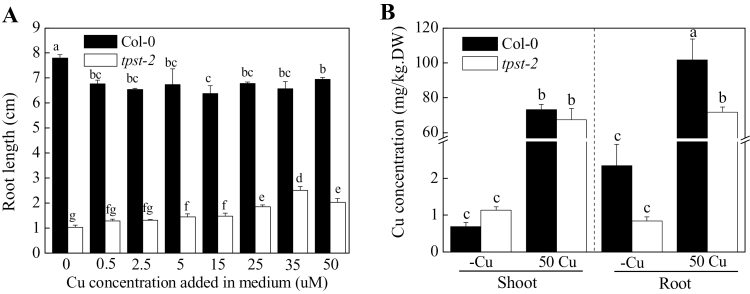
The *tpst-2* mutant plants are sensitive to Cu deficiency. (A) Root lengths of Col-0 and *tpst-2* mutant plants under various Cu concentrations. Plants were grown for 10 d on vertically placed MGRL medium. CuSO_4_ (0.5, 2.5, 5, 15, 25, 35, and 50 µM) was added to the medium (excluding -Cu medium). Letters represent significant differences at the 0.05 level based on Tukey’s test. Bar = SE, n=10. (B) Cu concentrations in roots and shoots of Col-0 and *tpst-2* mutant plants. Plants were grown for 11 d on vertically placed MGRL medium. CuSO_4_ (50 µM) was added to 50 Cu medium but not to -Cu medium. Letters represent significant differences at the 0.05 level based on Tukey’s test. Bar = SE, n = 10.

At increased concentrations, Cu induces the generation of harmful reactive oxygen species, which cause oxidative damage to cells at lipid, protein, and nucleic acid levels (Halliwell and Gutteridge, 1984; Drazkiewicz *et al.*, 2004). The seedlings of Col-0 and *tpst-2* were grown in various Cu conditions and root length was measured to further examine Cu sensitivity of *tpst-2* root elongation. The seedlings of Col-0 showed a longer root under -Cu and 50 Cu conditions than other Cu conditions (Fig. 2A). The root elongation of *tpst-2* was responsive to external Cu in a concentration-dependent manner (up to 35 µM Cu, referred to as 35 Cu); furthermore, 35 Cu elicited the strongest effect on the root elongation of *tpst-2* (Fig. 2A). By contrast, the addition of other divalent transition metals, such as Fe, Mn, Ni, and Zn, failed to recover normal *tpst-2* root elongation (Fig. S3). The root lengths of Col-0 and *tpst-2* were restrained under Fe deficiency, 50 µM Fe, and 100 µM Ni conditions (Fig. S3). In Cu treatment, CuSO_4_ was added to the media to increase the Cu and sulfate concentrations. CuCl_2_ was analysed to confirm that Cu was responsible for root elongation recovery of *tpst-2*. CuCl_2_ treatment elicited an effect on the root elongation of *tpst-2* similar to CuSO_4_ (Fig. 1A, Fig. S4); this result suggests that *tpst-2* is a mutant affecting Cu homeostasis.

### *TPST* mutation affects Cu homeostasis in *tpst-2* plants

Plants can sense internal and external Cu status and adapt to changing Cu conditions by modifying gene expression. Cu concentrations of *tpst-2* seedlings under -Cu and 50 Cu conditions were measured to determine whether Cu sensitivity of *tpst-2* mutant seedlings was affected. The results showed that *tpst-2* roots exhibited a lower Cu concentration than Col-0 under both the -Cu and 50 Cu conditions; however, this finding was not observed in the shoots. Thus, this result cannot be considered as a generalized observation (Fig. 2B). Under the -Cu condition, root Cu concentrations of *tpst-2* and Col-0 seedlings were 0.84±0.11 and 2.35±0.58mg/kg DW, respectively. Under the 50 Cu condition, root Cu concentrations of *tpst-2* and Col-0 were 71.9±2.9 and 102±12mg/kg DW, respectively (Fig. 2B). However, no significant difference in shoot Cu concentration was observed between Col-0 and *tpst-2* mutant plants grown under either -Cu or 50 Cu conditions (Fig. 2B). The altered root Cu concentration suggested that the *TPST* mutation is involved in Cu homeostasis.

**Fig. 3.**
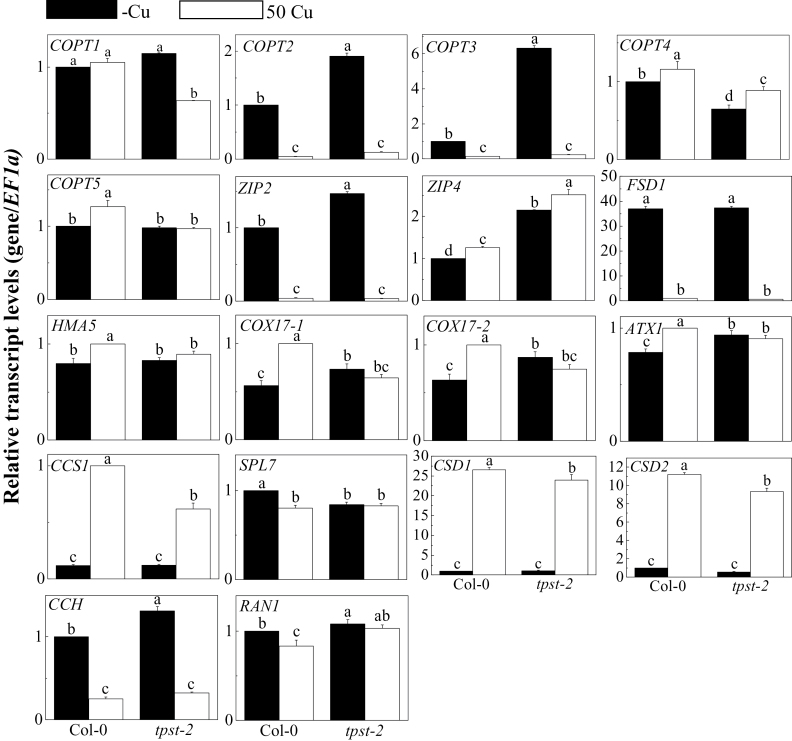
The expression levels of Cu-related genes in the root of Col-0 and *tpst-2* under -Cu/50 Cu conditions. Plants were grown for 10 d on vertically placed MGRL medium with or without 50 µM CuSO_4_. Total RNA was extracted from the whole roots of seedlings. At least 10 plants were used per replicate. The mRNA levels were normalized to the *EF1α* mRNA levels in the same samples. The data are expressed as means ± SE (n = 3, technical repeats) relative to the Col-0 value (defined as 1). Letters represent significant differences at the 0.05 level based on Tukey’s test.

To confirm this hypothesis, the mRNA levels of several genes involved in Cu homeostasis were determined in *tpst-2* mutant plants. This analysis included genes encoding Cu transporters (*COPT1*, *COPT2*, *COPT3*, *COPT4*, *COPT5*, *ZIP2*, *ZIP4*, and *RAN1*), Cu chaperones (*CCH*, *CCS*, *ATX*, *COX17-1*, *COX17-2*), -Cu indicators (*FSD1*, *CSD1*, *CSD2*), P-type ATPases (*HMA5*), and a master Cu regulator, *SPL7* (Fig. 3). Interestingly, the mRNAs of *COPT3* and *COPT2* accumulated to a considerably higher level (6.3-fold for *COPT3*, and 1.9-fold for *COPT2*) in *tpst-2* roots than in Col-0 roots under -Cu condition, although both mRNAs were upregulated in Col-0 and *tpst-2* roots by -Cu. The expression of *COPT1* was upregulated in *tpst-2* but remained unaffected in Col-0 roots by -Cu. Although the expression of *ZIP4* was not altered by -Cu in either Col-0 or *tpst-2* roots, *ZIP4* was constitutively expressed to a higher level in *tpst-2* than in Col-0. *COX17-1* and *COX17-2* expressions were downregulated in Col-0, but were not regulated in *tpst-2* roots by -Cu. Furthermore, the expressions of *SPL7*, *RAN1*, *ATX1*, *HMA5*, *COPT4*, and *COPT5* were not evidently upregulated by -Cu, even in Col-0 (Fig. 3). The expressions of *CCH*, *FSD1*, and *ZIP2* were upregulated by -Cu in both Col-0 and *tpst-2* roots (Fig. 3); by contrast, the expressions of *CSD1*, *CSD2*, and *CCS1* expression were downregulated by -Cu in both Col-0 and *tpst-2* roots (Fig. 3).

### *TPST* mutation increases ethylene production under -Cu condition

**Fig. 4.**
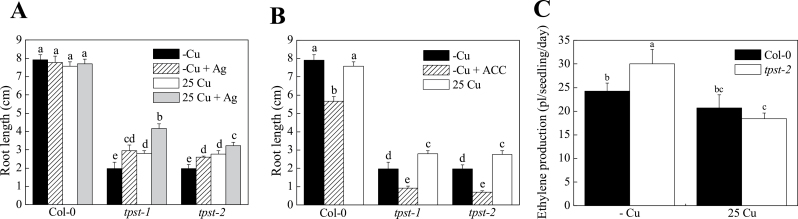
Ethylene measurement and response of *tpst-1* and *tpst-2* root to AgNO_3_ and ACC under different Cu conditions. (A) Combination treatment of plants with AgNO_3_ and Cu. Col-0, *tpst-1*, and *tpst-2* plants were grown for 14 d on vertically placed MGRL medium with or without additional 1 μM AgNO_3_. CuSO_4_ (25 µM) was added to 25 Cu medium but not to -Cu medium. Letters represent significant differences at the 0.05 level based on Tukey’s test. Bar = SE, n = 10. (B) Combination treatment of plants with ACC and Cu. Col-0, *tpst-1*, and *tpst-2* plants were grown for 14 d on vertically placed MGRL medium with or without additional 1 μM ACC. CuSO_4_ (25 µM) was added to 25 Cu medium but not to -Cu medium. Letters represent significant differences at the 0.05 level based on Tukey’s test. Bar = SE, n = 10. (C) Ethylene production of plants. Col-0 and *tpst-2* plants were grown for 5 d on vertically placed MGRL medium. CuSO_4_ (25 µM) was added to 25 Cu medium but not to -Cu medium. Letters represent significant differences at the 0.05 level based on Tukey’s test. Bar = SE, n = 30.

The role of Cu in the binding of ethylene to its receptors supports the role of Cu homeostasis in plant signalling and developmental regulation (Rodríguez *et al.*, 1999). In Cu transport across intracellular membranes, *RAN1* is a P-type ATPase that transports Cu to a late secretory compartment for delivery to ethylene receptors (Hirayama *et al.*, 1999). Considering that *TPST* is involved in Cu response, detailed study was performed to determine whether the disruption of *TPST* affects ethylene sensing and/or signalling. For this purpose, root elongation of Col-0 and *tpst-2* plants was determined under -Cu conditions with additional AgNO_3_ or ACC treatment, which are respectively known as an inhibitor of ethylene binding and a substrate of ACC oxidase, which makes ethylene (Lurssen *et al.*, 1979; Strader *et al.*, 2009). This result showed that the root elongation of *tpst-2* was responsive to AgNO_3_ and ACC treatment (Fig. 4A,B). Under -Cu conditions, *tpst-2* root elongation was partially rescued by additional AgNO_3_ treatment. Furthermore, the combination of AgNO_3_ and Cu elicited a greater effect on the root elongation of *tpst-2* (Fig. 4A). The response of *tpst-1* was similar to that of *tpst-2*. ACC also affected the root elongation of *tpst-2*, but this effect differed from that of AgNO_3_. With additional ACC treatment, *tpst-2* seedlings showed an 85% reduction in root length, whereas Col-0 showed a 42% reduction in root length (Fig. 4B). These results can be explained by the increased ethylene production in the *tpst-2* mutant and inhibition of the ethylene response by Cu and Ag.

To confirm this hypothesis, ethylene production was determined under various Cu conditions. Under normal Cu (1 µM) conditions, *tpst-2* and *tpst-1* exhibited higher ethylene production than Col-0 (Supplementary Fig. S5A). In addition, *tpst-2* yielded higher ethylene production than Col-0 under -Cu conditions; conversely, *tpst-2* displayed ethylene production similar to wild-type plants under 25 Cu conditions (Fig. 4C). These results confirmed that ethylene production by *tpst* mutants was higher than that by wild-type plants under -Cu conditions.

### *TPST* mutation affects genes associated with ethylene response, biosynthesis, and signal transduction

The expression of an ethylene-responsive marker gene was determined to further characterize the relationship between *TPST* and ethylene. Expression of the basic chitinase gene is induced by ethylene in adult *Arabidopsis* plants (Samac *et al*., 1990). In the present study, the basic chitinase gene exhibited a higher expression level in the roots of *tpst-2* and *tpst-1* under -Cu conditions than under 50 Cu conditions (Supplementary Fig. S5B). These results confirm that *TPST* mutation increases ethylene production.

**Fig. 5.**
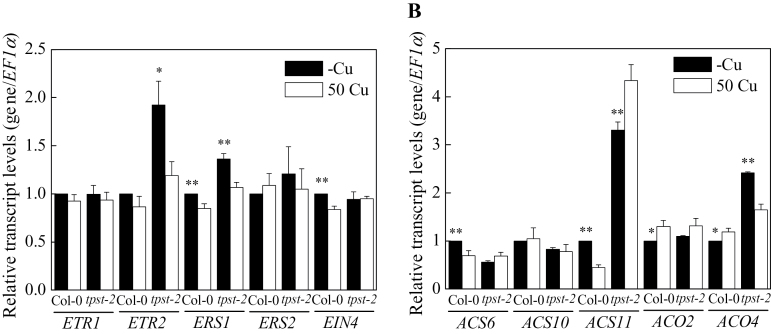
Expression of (A) ethylene receptor and (B) biosynthesis genes in roots of Col-0 and *tpst-2* by quantitative RT-PCR. Col-0 and *tpst-2* mutant plants were grown for 10 d on vertically placed MGRL medium. CuSO_4_ (50 µM) was added to 50 Cu medium, but not to -Cu medium. Total RNA was extracted from the whole roots of seedlings. At least 10 plants were used per replicate. The mRNA levels were normalized to those of *EF1α* in the same samples. The data are expressed as means ± SE (n = 3, technical repeats) relative to the Col-0 value (defined as 1). Asterisks represent significant differences from the 50 Cu conditions (**P < 0.01; *P < 0.05, Student’s t-test).

To examine how the *TPST* mutation disrupts ethylene production, the expression of several ethylene signal transduction and biosynthesis genes was examined by quantitative RT-PCR (Fig. 5) to investigate the mechanism by which *TPST* mutation disrupts ethylene production. Interestingly, the mRNA of *ACO4*, an ethylene biosynthesis gene, accumulated to a much higher level in the roots of *tpst-2* than in the roots of Col-0 under -Cu; by contrast, *ACO4* expression differed slightly between *tpst-2* and Col-0 under 50 Cu conditions. The ethylene receptor gene *ETR2* showed expression patterns similar to *ACO4*. This result suggests that *TPST* mediates the accumulation of high ethylene levels and induces the expression of signal transduction genes. However, only some of the genes associated with ethylene production and signal transduction were affected by *TPST*. The expression of *ACS11*, a biosynthesis gene, showed an opposite pattern between Col-0 and *tpst-2* under Cu treatment; in particular, *ACS11* was more highly expressed in *tpst-2* than in Col-0. A marked upregulation of other biosynthesis and signal transduction genes, including *ACS6*, *ACS10*, *ACO2*, *ETR1*, *ERS1*, *ERS2*, and *EIN4*, was not detected, even in wild-type roots (Fig. 5). These results suggest that *TPST* is involved in the regulation of some ethylene biosynthesis and signal transduction genes under -Cu.

### Expression of *TPST* is not regulated by ethylene or Cu

Ethylene is involved in the sensitivity of root elongation to -Cu in *tpst-2* mutants, so determining whether the expression of *TPST* is regulated by Cu or ethylene is important. To answer this question, *TPST* expression was measured under various Cu conditions combined with AgNO_3_ and ACC treatment. The quantitative RT-PCR results revealed that *TPST* transcript accumulation was not regulated by Cu, AgNO_3_, or ACC treatment (Supplementary Fig. S6).

### Treatment with the peptide hormone PSK suppresses ethylene production by *tpst-2* seedlings

**Fig. 6.**
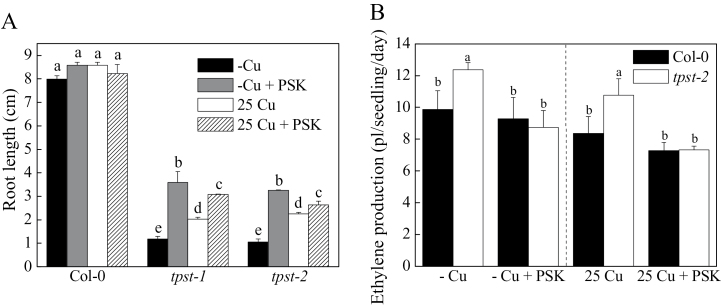
Root length and ethylene content measurement of Col-0 and *tpst-2* plants. (A) Ethylene content measurement of Col-0 and *tpst-2* plants. Col-0 and *tpst-2* plants were grown for 5 d on -Cu and 25 Cu MGRL medium with or without additional 10nM PSK. Letters represent significant differences at the 0.05 level using Tukey’s test. Bar = SE, n = 30. (B) Root elongation of *tpst-2* plants under Cu and PSK treatments. Col-0, *tpst-1*, and *tpst-2* plants were grown for 10 d on vertically placed MGRL medium with or without 10nM PSK. CuSO_4_ (25 µM) was added to 25 Cu medium, but not to -Cu medium. Letters represent significant differences at the 0.05 level using Tukey’s test. Bar = SE, n = 10.

*TPST* encodes tyrosylprotein sulfotransferases responsible for the sulfation of the peptide hormone PSK. The fact that ethylene production of *tpst-2* was elevated (Fig. 4C) suggests that PSK suppresses ethylene production in plants. To confirm this hypothesis, Col-0, *tpst-2*, and *tpst-1* plants were treated with 10nM PSK, and root length and ethylene production were measured. PSK treatment of *tpst-2* and *tpst-1* partially rescued root elongation (Fig. 6A). The extent of the rescue was considerably higher under -Cu than 25 Cu conditions. In addition, PSK treatment had a greater recovery effect on the root elongation of the *tpst* mutants than did Cu (Fig. 6A). Ethylene production by *tpst-2* plants decreased in the presence of PSK under -Cu or 25 Cu conditions, whereas ethylene production by Col-0 was unaffected by PSK treatment (Fig. 6B). These results suggest that PSK suppresses ethylene production in *tpst* mutants.

## Discussion

In the present study, *A. thaliana* mutants sensitive to -Cu conditions were identified and used to determine that *TPST* is involved in Cu-dependent root elongation (Fig. 1 and Supplementary Fig. S1). Furthermore, results demonstrate that *TPST* is involved in ethylene production and PSK suppresses ethylene production under -Cu conditions (Figs. 4 and 6). This represents a novel link between two phytohormones, PSK and ethylene, in a Cu-dependent manner.

Cell-to-cell communication is important for plants. Phytohormones (including auxin, cytokinins, ethylene, and brassinosteroids) and secreted peptides, which are a novel type of plant hormone, play critical roles in cell-to-cell communication in plants (Matsubayashi, 2011; Yamada and Sawa, 2013). In higher plants, many genes encoding small, secreted peptides have been identified (Matsubayashi, 2011). Modifications of these peptides, such as posttranslational sulfation of PSK, are important for their function (Komori *et al.*, 2009; Matsuzaki *et al.*, 2010; Zhou *et al.*, 2010).

In the present study, evidence was obtained that suggests a novel relationship between PSK and ethylene. The plant hormone ethylene influences various plant developmental processes, including germination, tissue senescence, fruit ripening, sex determination, and the response to a wide variety of stresses (Chang and Bleecker, 2004; Wu *et al.*, 2010). Ethylene is also part of the signalling pathway that modulates cell division in the quiescent centre in the stem cell niche during the postembryonic development of the root system (Ortega-Martínez *et al.*, 2007). In this study, several lines of evidence indicated that *TPST* suppresses ethylene production through the action of PSK (Figs. 4A,B and 6, and Supplementary Fig. S5). Based on these results and the hypotheses of this study combined with previous reports, a mechanism for the root elongation of *tpst-2* mutant plants is proposed. In wild-type *Arabidopsis* plants, active PSK is thought to repress ethylene production, which results in normal root elongation. The *tpst-2* mutant lacks the ability for the sulfation of the PSK precursor peptides, and ethylene production increases because of the lack of PSK. The high ethylene levels then decrease root elongation. Alternatively, since there is not a difference in ethylene production between *tpst-2* and Col-0 in all conditions, many sickly mutants and even WT plants subjected to stressful conditions (-Cu, in this case) may also overproduce ethylene, so it might also be reasonable that the *tpst* mutants have elevated levels of ethylene, and, thus, a subset of the *tpst* phenotypes is a consequence of enhanced ethylene signalling. Accordingly, it is also logical that silver ions can partially rescue some aspects of the *tpst* mutant phenotype that are caused by extra ethylene. Likewise, it is expectable that exogenous application of the peptide hormone PSK (the molecular target of the TPST-mediated enzymatic activity) can bypass the requirement for functional *TPST* and reduces the severity of the *tpst* mutant defects, thus lessening ethylene overproduction in this mutant.

Notably, root elongation of the mutant was partially rescued by high Cu (Fig. 1). Cu is required not only for ethylene binding but also for the signalling function of ethylene receptors. Cu likely downregulated ethylene signalling pathways in these experiments, rescuing root elongation by preventing the development of ethylene response phenotypes. Moreover, root elongation of the *tpst-2* mutant phenotype was partially recovered by AgNO_3_ (Fig. 4A), which could also affect auxin efflux independently of ethylene response (Strader *et al.*, 2009). Although ethylene production is affected by various stresses, and the link between PSK and ethylene may not be a direct link, the evidence suggests a new role of PSK in the regulation of ethylene production.

Cu is an essential micronutrient for normal growth and development, and deficiency of this metal has a severe effect on plant development. Thus, to respond to -Cu, plants have evolved cellular and molecular mechanisms to maintain Cu homeostasis. Despite the importance of preventing -Cu in plants, much remains to be elucidated about the mechanisms controlling Cu homeostasis and trafficking inside the cell.

Root length is a highly sensitive parameter frequently used to test plant Cu sensitivity (Sancenón *et al.*, 2004; Andrés-Colás *et al.*, 2006). A defect in Cu homeostasis dramatically affects root elongation in *Arabidopsis*. *Arabidopsis* seedlings defective in *COPT1* expression display a significant decrease in Cu acquisition and accumulation, and increased root length under Cu-limited conditions, whereas the roots could return to wild-type length when treated with 30 µM Cu sulfate (Sancenón *et al.*, 2004). The same situation has been observed with *hma5*, *atx1*, and *cch-atx1* double mutant (Andrés-Colás *et al.*, 2006; Shin *et al.*, 2012). The root length in 8-day-old seedlings is dramatically decreased in both *hma5* mutant alleles treated with 20 µM Cu, whereas no defect was observed in MS plates. Root length was shorter for *atx1* and *cch-atx1* than for the wild type and the *cch* mutant with excess Cu; additionally, with 25, 35, and 50 µM Cu, the root length was about 80%, 76%, and 57%, respectively, in the wild type. Moreover, *Arabidopsis* seedlings overexpressing *COPT1* or *COPT3* displayed increased endogenous Cu concentration, and they did not appreciably differ from the wild-type ones when they were grown on MS. However, the seedlings grown on 10 µM CuSO_4_ displayed dramatic inhibition in root growth.

The *tpst-2* mutant identified in this study showed a new and opposite phenotype to these observations mentioned above. The *tpst-2* mutant defective in *TPST* not only displayed a significant decrease in root Cu concentration (Fig. 2B), but also a significant decrease in root length under -Cu conditions. At the same time, the root elongation could be partially rescued by 50 µM Cu sulfate (Fig. 1). Notably, endogenous low Cu concentration in *tpst-2* mutant plants was perceived by the Cu sensor system, as suggested by the changes in the expression of the well-known Cu transporters *COPT2* and *COPT3* (Fig. 3). However, the Cu transcriptional factor *SPL7* mRNA level was not influenced by Cu in *tpst-2* mutant (Fig. 3). This difference in root elongation among *tpst-2* and other Cu-related mutants suggests that the effect of Cu on root elongation in *tpst-2*, which has point mutation of *TPST*, is different from the mechanisms of known Cu transporters (*COPT1* and *COPT3*) and chaperones (*ATX1* and *CCH*).

The present study was performed using a Cu response mutant. The finding that *tpst* mutants exhibit Cu responses and differential Cu accumulation in roots suggests a novel regulatory mechanism of Cu homeostasis, i.e., the involvement of PSK. The *tpst-2* mutant showed a significant decrease in root Cu concentration (Fig. 2B) and root length (Fig. 1) under -Cu conditions. Moreover, root elongation was partially rescued by 50 Cu (Fig. 1). Expression of the well-known Cu transporters *COPT2* and *COPT3* was higher in the mutant (Fig. 3). However, the mRNA level of the Cu transcriptional factor *SPL7* was unaffected by *tpst-2* mutation (Fig. 3). Such differences in the effect of *tpst-2* mutations among the Cu-inducible genes suggest that the roles of PSK in the Cu response are limited to Cu homeostasis or Cu sensing/signal transduction pathways. The *TPST* mRNA level was not regulated by Cu (Supplementary Fig. S6), suggesting that the relationship between PSK and Cu homeostasis/signalling is uni-directional. PSK affects Cu homeostasis/signalling, but Cu does not affect PSK.

At this time, it is not possible to demonstrate the mechanisms underlying PSK-mediated regulation of Cu homeostasis, but the data revealed a possible site of action. In the *tpst-2* mutant, the Cu concentration in roots was lower than that in the wild type (Fig. 2B), whereas expression of the Cu transporter genes *COPT2* and *COPT3* was significantly upregulated (Fig. 3). *Arabidopsis COPT3* proteins have been implicated in Cu transport (Sancenón *et al.*, 2004), and overexpression of *COPT3* increased the total content of Cu in the plant. COPT2 is a plasma membrane protein that functions in Cu acquisition and distribution, whose expression is regulated most strongly in -Cu (Sancenón *et al.*, 2003; Yamasaki *et al.*, 2009; del Pozo *et al.*, 2010). It is reasonable to assume that the roots of *tpst-2* mutants with high *COPT2* and *COPT3* expression have higher Cu concentration. However, the results shown in Fig. 2B contradict this hypothesis. Possibly, the *tpst-2* mutation affects the link between the high expression of *COPT2*/*3* and the high accumulation of Cu in the roots. PSK may affect the activity of *COPT2*/*3* and maintain the high *COPT2*/*3* expression and low Cu contents.

In summary, in the present study, a novel link between PSK and ethylene production has been identified, as well as a novel factor that regulates Cu homeostasis in *Arabidopsis*.

## Supplementary data

Supplementary data are available at *JXB* online.

**Table S1.** Primers used for RT-PCR and quantitative RT-PCR analyses.

**Fig. S1.** Mapping and cloning of the causal gene of Cu-deficiency-sensitive mutant *33–4*.

**(A)** Identification of the causal gene of *33–4* mutant by rough mapping and SOLiD. The left panel indicates rough mapping of the mutation. The markers and their positions are according to TAIR 10 database. The number of recombinants at each marker is indicated on the left side of the schematic chromosome. The right panel represents the result of sequencing analysis using SOLiD. Based on the analysis of the mapped region for SNP identification, the criterion is at least three reads with more than 60% point mutations. Using this criterion, two mutations were identified in the mapped region at positions 2448324 and 2491575 on chromosome 1 based on the TAIR 10 database.

**(B)** Schematic of the exon-intron structure of AT1G08030 based on the TAIR 10 database and the location of the point mutation (2491575) in *33–4* and T-DNA insertion in *tpst-1*. Boxes and lines represent exons and introns, respectively. White boxes represent 5′ and 3′ UTRs. The T-DNA is not drawn to scale. The point mutation disrupts splicing consensus from AG to AA.

**(C)** Genotyping of the mutant and transgenic plants. Col-0, *33–4*, *tpst-1*, F_1_ (*tpst-1* × *33–4*), and *35S:AtTPST*/*33–4* plants were grown for 10 d on vertically placed solid medium containing 50 µM CuSO_4_ (50 Cu) or no additional Cu (-Cu) medium. Bars = 1cm.

**Fig. S2.** The point mutation in the consensus splicing sequence of *TPST* fourth intron results in two different proteins. **(A)** RT-PCR analysis of *TPST* in Col-0 and *tpst-2*. M: 100-bp DNA ladder. **(B)** Original *TPST* gene structure. M: point mutation of *TPST* in *tpst-2* mutant plants; RT-F, RT-R and arrowheads indicate the positions and orientations of the primers used for RT-PCR. **(C)** Mutation in *TPST* intron splicing site results in two variant forms. The 1bp represents the first nucleic acid of the start codon (ATG) in the *TPST* sequence based on the TAIR 10 database.

**Fig. S3.** Root lengths of Col-0 and *tpst-2* seedlings under different metal conditions. The primary root length of 10-day-old seedlings grown in medium without Zn, Fe, Mn, Ni, or medium containing 50 or 100 µM of each element. The control values of root length were obtained in normal MGRL medium containing 1 µM Cu, 1 µM Zn, 8.6 µM Fe, and 10.3 µM Mn. The root lengths were expressed as means ± SE (n = 10).

**Fig. S4.** The response of *tpst-2* root elongation to CuCl_2._ Col-0 and *tpst-2* mutant plants were grown for 11 d on vertically placed MGRL medium with or without CuCl_2_ (50 µM). n = 10. Letters represent significant differences at the 0.05 level based on Tukey’s test.

**Fig. S5.** Ethylene production and expression of the ethylene marker gene basic chitinase. **(A)** Col-0, *tpst-1*, and *tpst-2* plants were grown on MS medium (1 μM Cu) for 5 d. Letters represent significant differences at the 0.05 level by Tukey’s test. Bar = SE, n = 30. **(B)** Quantitative RT-PCR analysis of basic chitinase expression in the roots of Col-0, *tpst-1*, and *tpst-2* seedlings. Col-0, *tpst-1*, and *tpst-2* plants were grown on vertically placed MGRL medium. CuSO_4_ (50 µM) was added to 50 Cu medium, but not to -Cu medium. Total RNA was extracted from the whole roots of 10-day-old seedlings. At least 10 plants were used per replicate. Levels of basic chitinase mRNA were normalized to those of *EF1α* in the same samples. The data were expressed as means ± SE (n = 3, technical repeats) relative to the Col-0 (-Cu) value (defined as 1). Asterisks represent significant differences from the 50 Cu condition (**P < 0.01, Student’s t-test).

**Fig. S6.** The expression pattern of *TPST* under various Cu conditions with or without AgNO_3_ and ACC treatment. Col-0 plants were grown for 10 d on vertically placed MGRL medium with -Cu or various Cu concentrations (1, 25, 35, and 50 µM). Next, 1 μM AgNO_3_ and 1 μM ACC were added to the 1 Cu medium. Total RNA was extracted from the whole roots of seedlings. At least 10 plants were used per replicate. Levels of *TPST* mRNA were normalized to those of *EF1α* in the same samples. The data were expressed as means ± SE (n = 3, technical repeats) relative to the value of Col-0 plants grown on 1 Cu medium (defined as 1). No significant differences were found based on Tukey’s test.

Supplementary Data
