# Supplementary material for: An Arabidopsis thaliana copper-sensitive mutant suggests a role of phytosulfokine in ethylene production
Source: J Exp Bot. 2015 Apr 23;66(13):3657–67. doi: 10.1093/jxb/erv105 (PMC4473973; doi:10.1093/jxb/erv105)
Supplement: Supplementary Data [file supp_66_13_3657__index.html]

An Arabidopsis thaliana copper-sensitive mutant suggests a role of phytosulfokine in ethylene production — Supplementary Data 

# An *Arabidopsis thaliana* copper-sensitive mutant suggests a role of phytosulfokine in ethylene production

## Supplementary Data

Data files

**Files in this Data Supplement:**

- Supplementary Data - Supplementary Data
