# Supplementary material for: An Arabidopsis thaliana copper-sensitive mutant suggests a role of phytosulfokine in ethylene production
Source: J Exp Bot. 2015 Apr 23;66(13):3657–67. doi: 10.1093/jxb/erv105 (PMC4473973; doi:10.1093/jxb/erv105)
Supplement: Supplementary Data [file supp_erv105_jexbot145318_file001.pdf]

An *Arabidopsis thaliana* copper-sensitive mutant suggests a role of phytosulfokine in ethylene production. *Tao Wu, Takehiro Kamiya, Hiroko Yumoto, Naoyuki Sotta, Yamaguchi Katsushi, Shuji Shigenobu, Yoshikatsu Matsubayashi and Toru Fujiwara*

Table S1. Primers used for RT-PCR and qRT-PCR analyses

**Gene**	**Forward primer (5'-3')**	**Reverse primer (5'-3')**
COPT1	GTTAATCCAAACCGCCGTGTA	GCCAGAGCGACGAGAAACAC
TPST RT-PCR	AAGGAAAAGTGCAAGTTGTTAGC	TGTGGATGATGTCATGAGCAGT
COPT2	TTGGGGTAAGAACACGGAGGT	TGACACGTAGGATCGGTGAATG
COPT3	CACCATCATCGTTCTTCCAACA	CGGCGAGACAGACCCAATAC
COPT4	GGGATGTATGCACTCGCACTTA	GCTTATCGGCACCCTGTTTG
COPT5	GAGAATCGCCGCATCCAAT	TGACGCCGAAAAGAAGAACC
ZIP2	ACGTTGCGGTTAACCATCTC	CGAGGAAGACGGCAATAAAC
ZIP4	TCACCATAGGCATAGTCACTC	AATCCCGAGCTCCAATATCTG
FSD1	TCGGCTCTTTCCCATTGCTT	TGGTCTTCGGTTCTGGAAGTCA
HMA5	GGGAAGCCCGTTGTTGTGA	CCTTTGCTAACGGATGCTCACT
COX17-1	GATTGATTCCTCCACCCACTTC	CATATCCTCTTCTTTGGTTTCGTC
COX17-2	AGACAGTGCGTGTTCTTTGGAC	AAGCGGATTCACCGTGTTCTA
ATX1	TGTTCCAAGCCGTATCCTATCA	TCCACGCCTTCCATTTTCC
CCS1	GCAAACTGGTCGAAAAGCTC	GTCAGGGCCTTTGAATTCTG
SPL7	GAGCTGGAGGGCTATATCCG	ACAGTCATCGAGCCTCTTCC
CDS1	CCCTGAGGATGCTAATCGACAT	TGGCAATCAGTGATTGTGAAGG
CSD2	CATGACACACGGAGCTCCAG	CACCCTTTCCGAGGTCATCC
CCH	CGTTGTCCTCAAAGTTGGTATGTC	CCTTTCACTGTCACCTTTTGCTC
RAN1	TTACCCAGACGAATGGCTTC	TTGCCACCATAACAGCAGTC
ETR1	GAATGGTGACGAAGGGACTT	ACTCGGAGACACTCCTCGTT
ETR2	TTATGAAGTGGCCATGAGGA	GCACACTTGTCCCACATTTC
ERS1	AGATGCGTTGATTGCTCTTG	GAAATGCGTAGCTCCACAGA
ERS2	AATGAGGAGAACCAGCGAGT	GCTGACTCTTTCACAACCGA
EIN4	CTCTGCTGTTTGGATGCCTA	TCGGGATTACCCTGAAACTC
ACS6	TCCTGACCGGATTGTTATGA	ATTCACTCCGGTTCTCCATC
ACS10	GGCTTAGATCCGCTGCTATC	GGGTTGGAGATGAGACAGGT
ACS11	CAAACCCACTTGGAACCTCT	TGGTGAACTCAGGAGAGTCG
ACO2	AGGTGATAACCAACGGGAAG	CTCGACAAGCGAAGTAGCTG
ACO4	ATGGGATTTCACTCGAGCTT	GTCGTTGACTTCAGAGCGAA
TPST	TTAACTTCTGCGTCAAGG	AAAGGCATAAGCATCTCC
basic chitinase	TTTATCACCGCTGCAAAGTC	CATGAATATGGTCCGTCTGG
EF1α	CCTTGGTGTCAAGCAGATGA	TGAAGACACCTCCTTGATGATTT
CAPS	AAGGAAAAGTGCAAGTTGTTAGC	TGTGGATGATGTCATGAGCAGT

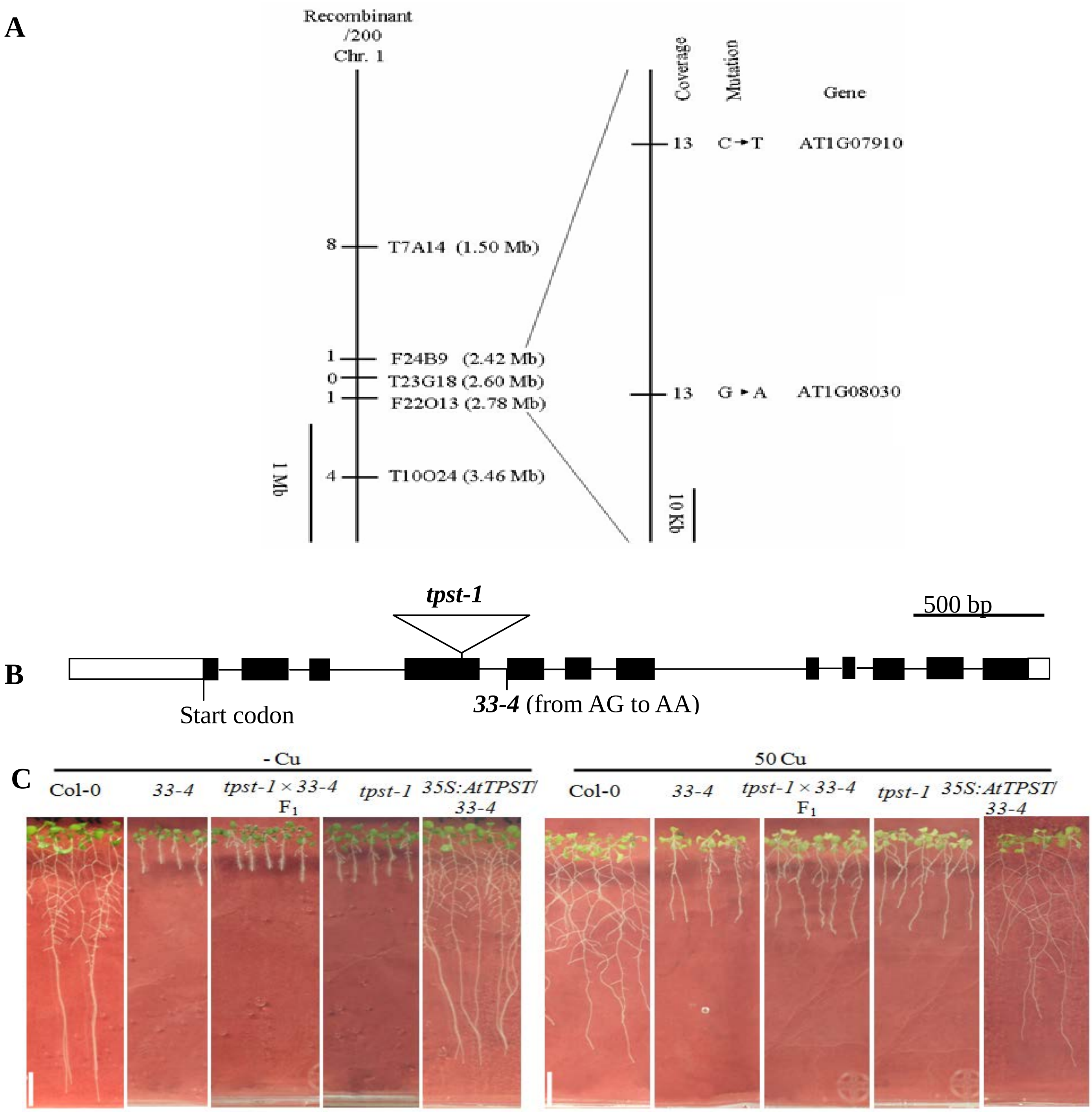

Figure S1. Mapping and cloning of the causal gene of Cu deficiency sensitive mutant *33-4*.

(A) Identification of the causal gene of *33-4* mutant by rough mapping and SOLiD. The left panel indicates rough mapping of the mutation. The markers and their positions are according to TAIR 10 database. The number of recombinants at each marker is indicated on the left side of the schematic chromosome. The right panel represents the result of sequencing analysis using SOLiD. Based on the analysis of the mapped region for SNP identification, the criterion is at least three reads with more than 60% point mutations. Using this criterion, two mutations were identified in the mapped region at positions 2448324 and 2491575 on chromosome 1 based on the TAIR 10 database.

(B) Schematic of the exon-intron structure of AT1G08030 based on the TAIR 10 database and the location of the point mutation (2491575) in *33-4* and T-DNA insertion in *tpst-1*. Boxes and lines represent exons and introns, respectively. White boxes represent 5’ and 3’ UTRs. The T-DNA is not drawn to scale. The point mutation disrupts splicing consensus from AG to AA.

(C) Genotyping of the mutant and transgenic plants. Col-0, *33-4*, *tpst-1*, F_1 (*tpst-1* × *33-4*), and *35S:AtTPST*/*33-4* plants were grown for 10 d on vertically placed solid medium containing 50 µM $CuSO_4$ (50 Cu) or no additional Cu (-Cu) medium. Bars = 1 cm.

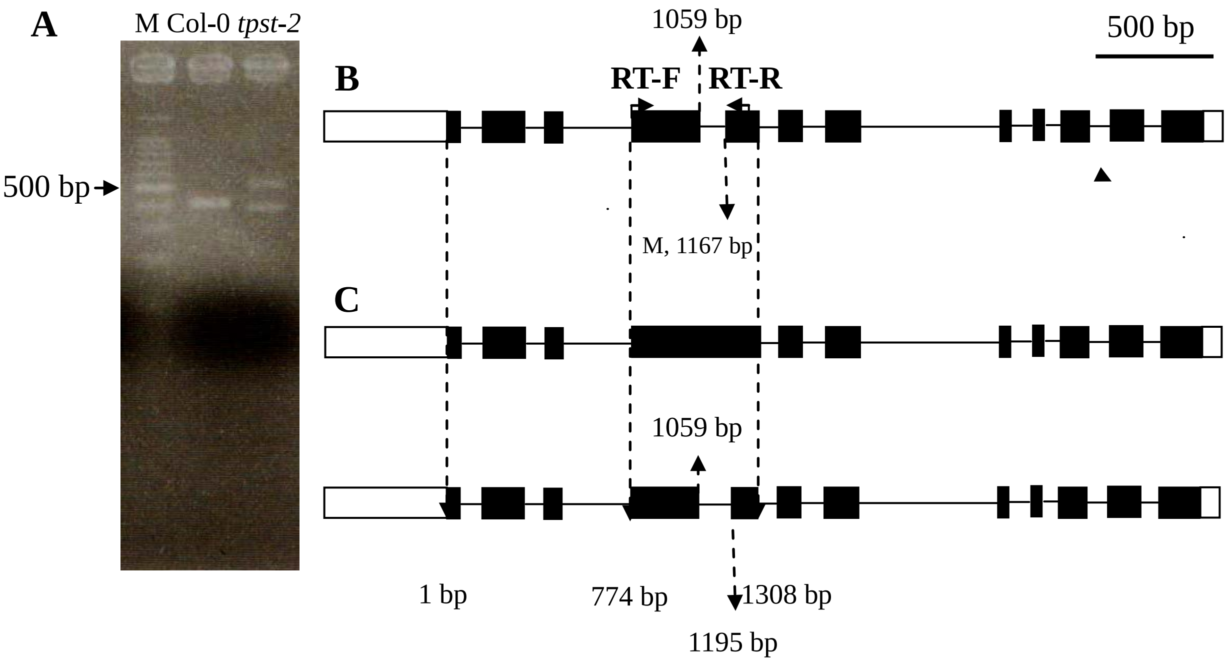

Figure S2. The point mutation in the consensus splicing sequence of *TPST* fourth intron results in two different proteins. (A) RT-PCR analysis of *TPST* in Col-0 and *tpst-2*. M: 100-bp DNA ladder. (B) Original *TPST* gene structure. M: point mutation of *TPST* in *tpst-2* mutant plants; RT-F, RT-R and arrowheads indicate the positions and orientations of the primers used for RT-PCR. (C) Mutation in *TPST* intron splicing site results in two variant forms. The 1 bp represents the first nucleic acid of the start codon (ATG) in the *TPST* sequence based on the TAIR 10 database.

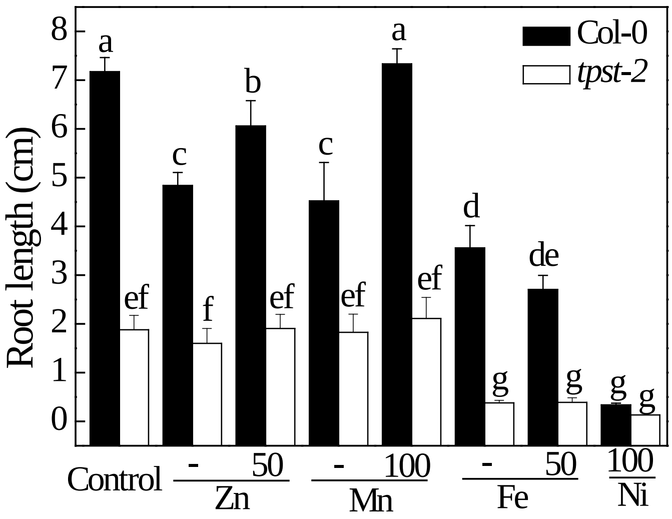

Figure S3. Root lengths of Col-0 and *tpst-2* seedlings under different metal conditions. The primary root length of 10-d-old seedlings grown in medium without Zn, Fe, Mn, Ni or medium containing 50 or 100 µM of each element. The control values of root length were obtained in normal MGRL medium containing 1 µM Cu, 1 µM Zn, 8.6 µM Fe, 10.3 µM Mn. The root lengths were expressed as means ± SE (n = 10).

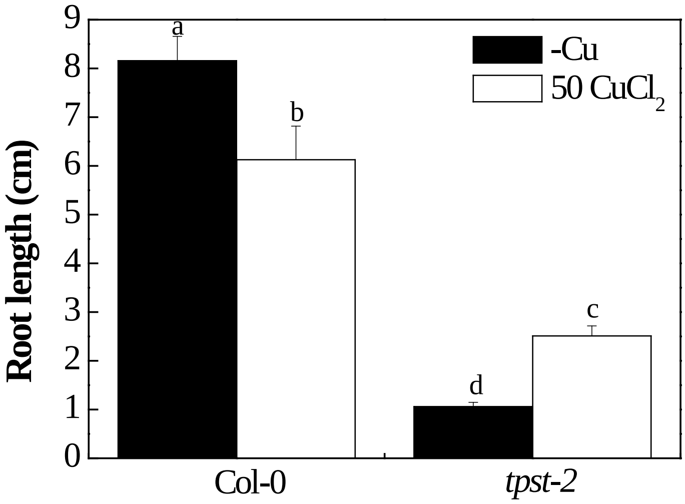

Figure S4. The response of *tpst-2* root elongation to $CuCl_2$. Col-0 and *tpst-2* mutant plants were grown for 11 d on vertically placed MGRL medium with or without $CuCl_2$ (50 µM). n = 10. Letters represent significant differences at the 0.05 level based on Tukey's test.

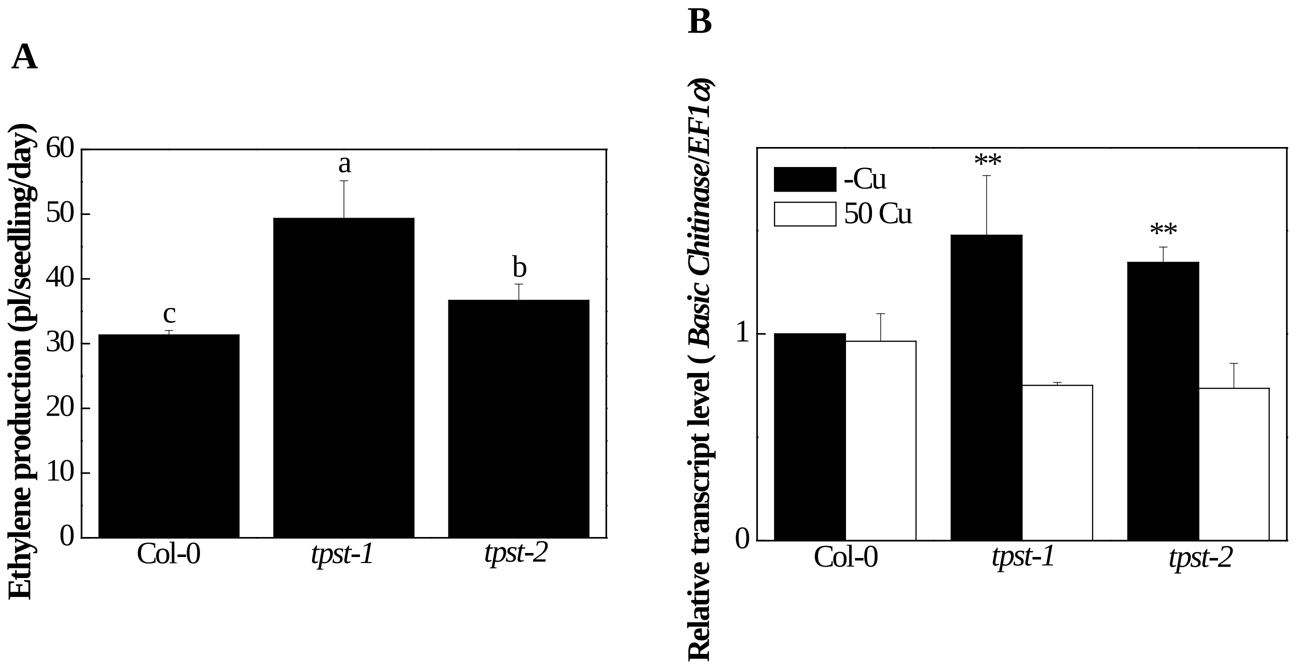

Figure S5. Ethylene production (A) and expression of the ethylene marker gene *basic chitinase* (B). (A) Col-0, *tpst-1*, and *tpst-2* plants were grown on MS medium (1 µM Cu) for 5 d. Letters represent significant differences at the 0.05 level by Tukey's test. Bar = SE, n = 30. (B) qRT-PCR analysis of *basic chitinase* expression in the roots of Col-0, *tpst-1*, and *tpst-2* seedlings. Col-0, *tpst-1*, and *tpst-2* plants were grown on vertically placed MGRL medium. $CuSO_4$ (50 µM) was added to 50 Cu medium, but not to - Cu medium. Total RNA was extracted from the whole roots of 10-d-old seedlings. At least 10 plants were used per replicate. Levels of *Basic Chitinase* mRNA were normalized to those of *EF1α* in the same samples. The data were expressed as means ± SE (n = 3, technical repeats) relative to the Col-0 (-Cu) value (defined as 1). Asterisks represent significant differences from the 50 Cu condition (**P < 0.01, Student's t-test).

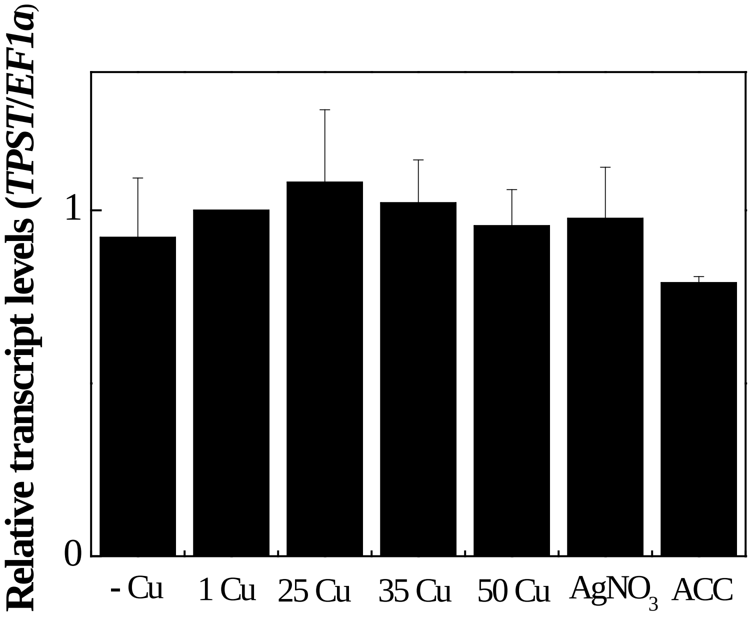

Figure S6. The expression pattern of *TPST* under various Cu conditions with or without $AgNO_3$ and ACC treatment. Col-0 plants were grown for 10 d on vertically placed MGRL medium with -Cu or various Cu concentrations (1, 25, 35, and 50 μM). Next, 1 μM $AgNO_3$ and 1 μM ACC were added to the 1 Cu medium. Total RNA was extracted from the whole roots of seedlings. At least 10 plants were used per replicate. Levels of *TPST* mRNA were normalized to those of *EF1α* in the same samples. The data were expressed as means ± SE (n = 3, technical repeats) relative to the value of Col-0 plants grown on 1 Cu medium (defined as 1). No significant differences were found based on Tukey's test.
